# Metagenomic sequencing, molecular characterization, and Bayesian phylogenetics of imported type 2 vaccine-derived poliovirus, Spain, 2021

**DOI:** 10.3389/fcimb.2023.1168355

**Published:** 2023-05-02

**Authors:** Maria Dolores Fernandez-Garcia, Martin Faye, Francisco Diez-Fuertes, Antonio Moreno-Docón, Maria Dolores Chirlaque-López, Ousmane Faye, Maria Cabrerizo

**Affiliations:** ^1^ Enterovirus and Viral Gastroenteritis Unit/National Polio Laboratory, National Centre for Microbiology, Instituto de Salud Carlos III, Madrid, Spain; ^2^ Consortium of Epidemiology and Public Health (CIBERESP), Instituto de Salud Carlos III, Madrid, Spain; ^3^ Virology Department, Institut Pasteur de Dakar, Dakar, Senegal; ^4^ AIDS Immunopathogenesis Unit, Instituto de Salud Carlos III, Madrid, Spain; ^5^ Consortium of Infectious Diseases, Instituto de Salud Carlos III, Madrid, Spain; ^6^ Microbiology Department, Hospital U. Virgen de la Arrixaca, Murcia, Spain; ^7^ Instituto Murciano de Investigación Biosanitaria (IMIB)-Arrixaca, Murcia University, Murcia, Spain; ^8^ Department of Epidemiology, Murcia Regional Health Council, Murcia, Spain

**Keywords:** vaccine-derived polio virus, whole-genome, metagenomics, recombination, molecular epidemiology, OPV (oral polio vaccine), Bayesian phylogenetic analysis

## Abstract

**Introduction:**

In 2021, a type 2 vaccine-derived poliovirus (VDPV2) was isolated from the stool of a patient with acute flaccid paralysis (AFP) admitted to Spain from Senegal. A virological investigation was conducted to characterize and trace the origin of VDPV2.

**Methods:**

We used an unbiased metagenomic approach for the whole-genome sequencing of VDPV2 from the stool (pre-treated with chloroform) and from the poliovirus-positive supernatant. Phylogenetic analyses and molecular epidemiological analyses relying on the Bayesian Markov Chain Monte Carlo methodology were used to determine the geographical origin and estimate the date of the initiating dose of the oral poliovirus vaccine for the imported VDPV2.

**Results:**

We obtained a high percentage of viral reads per total reads mapped to the poliovirus genome (69.5% for pre-treated stool and 75.8% for isolate) with a great depth of sequencing coverage (5,931 and 11,581, respectively) and complete genome coverage (100%). The two key attenuating mutations in the Sabin 2 strain had reverted (A481G in the 5’UTR and Ile143Thr in VP1). In addition, the genome had a recombinant structure between type-2 poliovirus and an unidentified non-polio enterovirus-C (NPEV-C) strain with a crossover point in the protease-2A genomic region. VP1 phylogenetic analysis revealed that this strain is closely related to VDPV2 strains circulating in Senegal in 2021. According to Bayesian phylogenetics, the most recent common ancestor of the imported VDPV2 could date back 2.6 years (95% HPD: 1.7–3.7) in Senegal. We suggest that all VDPV2s circulating in 2020–21 in Senegal, Guinea, Gambia, and Mauritania have an ancestral origin in Senegal estimated around 2015. All 50 stool samples from healthy case contacts collected in Spain (n = 25) and Senegal (n = 25) and four wastewater samples collected in Spain were poliovirus negative.

**Discussion:**

By using a whole-genome sequencing protocol with unbiased metagenomics from the clinical sample and viral isolate with high sequence coverage, efficiency, and throughput, we confirmed the classification of VDPV as a circulating type. The close genomic linkage with strains from Senegal was consistent with their classification as imported. Given the scarce number of complete genome sequences for NPEV-C in public databases, this protocol could help expand poliovirus and NPEV-C sequencing capacity worldwide.

## Introduction

Poliomyelitis is caused by poliovirus, a human enterovirus (family *Picornaviridae*, genus *Enterovirus*, species *Enterovirus C*). Since the Global Polio Eradication Initiative (GPEI) was launched in 1988, polio cases have been reduced by more than 99% (Global Polio Eradication Initiative). The GPEI managed to eradicate wild poliovirus (WPV) type 2 in 2015 and type 3 in 2019. The main strategies for eradication were mass immunization with the oral poliovirus vaccine (OPV) and surveillance of acute flaccid paralysis (AFP) cases and their contacts (Global Polio Eradication Initiative). OPV is a live-attenuated vaccine (with the three Sabin poliovirus serotypes) that replicates in the gut for a short time. Here, parental Sabin strains can mutate and be excreted into the environment ***via*** feces (Kew et al., 2005). Therefore, if polio vaccine coverage is low in a community and sanitation is inadequate, the mutated viruses may be transmitted in unimmunized and under-immunized populations, leading to genetic drift and the emergence of new pathogenic strains known as circulating vaccine-derived polioviruses (cVDPVs) (Kew et al., 2005). VDPVs currently constitute a major challenge to the polio eradication endgame, given their potential to cause paralysis and/or persistent circulation in human communities. Because most cVDPVs are type 2, in April 2016, a global switch from the trivalent oral polio vaccine to a bivalent oral polio vaccine containing only types 1 and 3 was implemented to remove all live-attenuated type 2 vaccines and their associated risk. The switch and delays in introducing a dose of inactivated polio vaccine (IPV) into essential immunization in all regions led to decreasing immunity to type 2 poliovirus (PV-2) (World Health Organization, 2021a). Decreasing immunity, together with the use of monovalent vaccine OPV2 in outbreak control campaigns, failures to control movements of VDPVs between countries, and difficulties in maintaining adequate vaccination coverage due to COVID-19, all resulted in a rapid growth of cVDPV2 outbreaks, with more cases of cVDPV2 than WPV1 annually since 2017 (Alleman et al., 2021; World Health Organization, 2021a).

If poliomyelitis is not eradicated globally, the risk of the virus being reintroduced in Europe remains. Two EU/EEA countries (Poland and Romania) and one neighboring country (Ukraine) are considered at risk of sustained poliovirus transmission following WPV importation or the emergence of cVDPV due to significant gaps in population immunity (World Health Organization, 2021b). In Spain, OPV was replaced by IPV in 2004, and national coverage of three doses in children’s first year of age exceeds 95% (Ministry of Health, Social Services and Equality, 2023). The National Action Plan for Poliomyelitis Eradication (NAPPE) was first established in 1998, and the study of AFP cases is the cornerstone of poliovirus surveillance, along with non-polio enterovirus (NPEV) surveillance (Masa-Calles et al., 2018). Since the OPV was replaced, only two VDPV have been identified, one in 2005 and the other in 2019 (Avellón et al., 2008; Informe anual, 2019). Both VDPVs were imported and isolated from immunocompromised patients. However, in September 2021, an imported paralysis case from Senegal was notified in Spain, identifying VDPV2 in a stool sample of the patient. All measures taken and actions carried out were within the framework of the NAPPE and the recommendations issued under the ongoing Public Health Emergency of International Concern on the risk of international spread of poliovirus (López et al., 2021). Molecular characterization of poliovirus isolates detected through AFP and environmental surveillance activities is key to distinguishing vaccine-related from WPV, and identifying genetic linkages between isolates to identify patterns of circulation (Classification and reporting of vaccine-derived polioviruses (VDPV), 2016). This molecular characterization is routinely based on Sanger sequencing of the viral protein 1 (VP1) capsid region. However, surveillance of poliovirus based on the whole genome has proven an essential tool for the final stages of polio eradication (Montmayeur et al., 2017). In this report, we describe the virological study of the case based on the VP1 and the whole-genome analyses of the detected VDPV2.

## Materials and methods

### The AFP case

Briefly, a child under the age of 6 years from Senegal traveled to Murcia, in the southeast of Spain, on August 2021, to be admitted to the hospital to continue treatment of an AFP with unknown origin and onset of symptoms in July 2021 in Senegal. The child was discharged from the hospital after showing clinical improvement. The diagnosis was acute anterior meningomyeloradiculitis because of an enterovirus infection (coxsackievirus B4 was detected in a respiratory sample during hospitalization). The child stayed with a local family until September 2021, then returned to Senegal. The case’s vaccination card showed four doses of OPV (received in the first year of life; last OPV dose: August 2016), followed by one dose of IPV. All of them were received during the first year of age. The case had no evidence of primary immunodeficiency. The AFP case was notified retrospectively in September, and a stool sample collected in August was sent to the National Polio Laboratory (NPL) of the National Centre for Microbiology (CNM) for a poliovirus study (López et al., 2021).

### Virological methods

The AFP case was laboratory investigated according to the isolation assay algorithm recommended by WHO (World Health Organization, 2004a) and the Spanish NAPPE (Ministerio de Sanidad, Asuntos Sociales e Igualdad, 2016). In addition to the single sample from the case, two stool samples from 25 healthy close contacts of the patient were examined. Analysis of the presence of poliovirus in all samples was performed by culture on RD and L20B cell lines following the 14-day algorithm (World Health Organization, 2004a; World Health Organization, 2004b). In addition, real-time RT-PCR (Cabrerizo et al., 2014) was used for the detection of enterovirus/poliovirus in clinical samples. Four RT-nested PCRs on the 3’VP1 region (specific for enterovirus species A to D) followed by sequencing were applied subsequently to those enterovirus-positive samples or isolates for genotyping (Cabrerizo et al., 2008). Finally, characterization of the detected poliovirus (WPV, OPV, or VDPV) was carried out using intratypic differentiation (ITD) assays (Gerloff et al., 2018), as was amplification and sequencing of the complete VP1 region of the viral genome. In Senegal, 25 contacts of the AFP case were studied by the WHO-accredited reference polio laboratory at the Institut Pasteur of Dakar, as previously described (Faye. et al., 2022).

### Wastewater samples

The presence of poliovirus was also analyzed in four raw wastewater samples collected at two inlet points of the wastewater treatment plant from the area where the case stayed on two different days (15 and 20 September). The same culture and molecular methods applied to clinical samples were used. Samples had been processed and concentrated previously using a protocol based on precipitation with AlCl_3_ (Randazzo et al., 2020).

### Metagenomic sequencing and bioinformatic analysis

Full-genome sequences of cVDPV2 from the AFP case were obtained from fecal suspension and from the L20B-positive supernatant. Fecal samples were prepared according to WHO standard procedures (World Health Organization, 2004a). Briefly, fecal samples were pre-treated with chloroform, to which enteroviruses are resistant, to remove bacteria and fungi. This was clarified at 4,000 rpm for 20 min at 4°C, contributing to the removal of host cellular debris and bacteria. This fecal suspension was used to inoculate cell cultures. Culture supernatants were frozen, thawed three times, and clarified. RNA was extracted from both stool-clarified suspension and clarified supernatant using the Quick RNA viral kit (Zymo). Sample library preparation was conducted using the NEBNext Ultra II Directional RNA Library Prep Kit for Illumina with NEBNext Multiplex Oligos for Illumina Set 3 (New England BioLabs Inc., USA) from a total concentration of 10 ng of RNA quantified using the QuantiFluor RNA System (E3310, Promega). Both libraries were sequenced using an Illumina MiSeq Reagent Kit v2 Nano on a MiSeq sequencer (300 cycles). The resulting data was analyzed using PikaVirus, a NextFlow pipeline openly available at https://github.com/BU-ISCIII/PikaVirus, and consisted of the following steps: quality control of the samples using FastQC (v. 0.11.9) and MultiQC (v. 1.9) for quality assessment, and FastP (v. 0.20.1) for trimming; potential viral genomes identification within the trimmed reads using MASH screening (v. 2.3) against the full GenBank viral assemblies database (release 248, downloaded on 2 April 2022); mapping of potentially present viral genomes against the samples using BowTie2 (v. 2.4.4); and obtention of coverage statistics using SamTools (v. 1.12); and BedTools (v. 2.30.0). Human host reads were subtracted by performing a kmer-based mapping of the trimmed reads with the GRCh38 NCBI human genome using Kraken2 v.2.1.2 (Wood et al., 2019). Reads that aligned to the human genome were excluded from further analysis. Spades v3.14.0 in *rnaviral* mode was used to perform a *de novo* assembly of non-host reads. Contigs taxonomic annotation was based on alignment to NCBI’s viral database using BLAST v2.9.0+. Results were visualized using Krona. For viral consensus genome reconstruction, we used the viralrecon pipeline v2.5 (https://github.com/nf-core/viralrecon) written in Nextflow (https://www.nextflow.io/) in collaboration with the nf-core (https://nf-co.re/) community and the Bioinformatics Unit of the Institute of Health Carlos III (BU-ISCIII) (https://github.com/BU-ISCIII) (Patel et al). The GenBank accession number of the isolate complete genome sequence is OQ401876.

### Phylogenetic and recombination analysis

Phylogenetic trees were constructed by the neighbor-joining method with 1,000 bootstrap replicates using Molecular Evolutionary Genetic Analysis (MEGA version 10) software (https://www.megasoftware.net/). We used published VP1 coding sequences available in GenBank, retrieved in November 2022. Nucleotide (nt) sequence alignment was performed by the ClustalW multiple alignment program within the BioEdit Sequence Alignment Editor package. Similarity plot analysis was performed by using the Simplot program, v3.5.1 with the Kimura distance model and a window size of 400 bp moving in 40 nt steps with 1,000 resamplings.

### Estimation of dates of the initiating OPV dose

The date and location of the OPV that initiated the emergence of the SPA866 case were estimated using a Bayesian Markov chain Monte Carlo (MCMC) approach. Root-to-tip genetic distances against sample collection dates were examined in a preliminary analysis with TempEst v.1.5.3 to identify the temporal signal of the VP1 nt sequence data. The Bayesian analysis was performed with BEAST software v1.10.4 (Rambaut et al., 2016) using the nt-substitution model described by Shapiro et al. (2006) with a discrete gamma distribution to accommodate rate variation among sites in the alignment and allowing different rates of substitution for the first plus second versus the third codon position. Lineage-specific rate heterogeneity was taken into account by using the uncorrelated relaxed molecular clock model with a lognormal rate distribution (Drummond et al., 2005). A Bayesian Skyline coalescent model assuming that a small random sample from a large population is included in the data set was selected. Two independent MCMC runs of 100 million states sampling every 10,000 steps were computed and mixed. The convergence of MCMC chains was checked using Tracer v.1.7.1, ensuring that the effective sample size values were greater than 200 for each estimated parameter (Rambaut et al., 2018). The maximum clade credibility trees were obtained from the tree posterior distribution using TreeAnnotator v.1.10.4 after a 10% burn-in. Phylogenetic trees were visualized and annotated with FigTree v.1.4.4 to indicate the mean estimated values and 95% highest posterior density (HPD) intervals for OPV dose dates and the posterior probability values for the most probable location.

## Results

### The AFP case: Poliovirus detection and initial molecular characterization of the stool isolate

The poliovirus was identified first by the presence of a cytopathic effect on both L20B and RD cell lines. Viral RNA extracted directly from stool suspension and from L20B-positive supernatant tested positive for enterovirus by real-time RT-PCR and RT-nested PCR. Genotyping in the 3’-VP1 region indicated that the enterovirus was a PV-2. The ITD assays, performed simultaneously at the CNM and at the Regional Polio Laboratory at the Robert Koch Institute (RKI) in Berlin, confirmed it was a PV-2 (both in the L20B-positive supernatant and in the stool sample). The isolate is hereinafter referred to as SPA866. Sanger sequencing of the complete VP1 region (903 nt) of the PV-2 detected in the L20B supernatant was performed in CNM and RKI simultaneously with identical results. The complete VP1 coding sequence differed from Sabin 2 (AY184220) by 5.1% (46 nt changes), confirming its classification as VDPV-2 according to WHO-adopted criteria (>0.6% genetic divergence in the complete genomic region) (Classification and reporting of vaccine-derived polioviruses (VDPV), 2016). Among the seven amino acid changes found in the VP1, one was the Ile143Thr substitution (encoded by the U_2909_ → C nt mutation), which represents the reversion of a key attenuation determinant of Sabin 2 (Ren et al., 1991).

### Whole-genome sequencing of the detected VDPV-2

Complete genomic sequencing of strain SPA866 was performed using a sequence-independent metagenomic approach from the pre-treated stool suspension and from the L20B-positive supernatant. For the stool suspension, a total of 439,714 reads were obtained. Of these, the percentage of viral reads that mapped to the targeted poliovirus genome accounted for 69.5% (n = 305,565), with a median deep coverage of 5,931 and a coverage >10× of 100%. For the isolate, a total of 777,948 reads were obtained. Of these, viral reads mapped to the poliovirus genome accounted for 75.8% (n = 589,645), with a median deep coverage of 11,581 and a coverage >10× of 100%. Performing *de novo* assembly of reads, no coinfecting pathogens were detected in the samples (only commensal bacteria of Mycoplasma spp.). Metagenomics in both samples yielded the same full-genome VDPV-2 sequence. The VP1 Sanger sequence described in the previous section was identical to that obtained by metagenomic sequencing. The consensus sequence was 7,427 nt in length, including a 5´ untranslated region (UTR) of 738 nt, a single open reading frame (ORF) of 6621 nt encoding a single polyprotein of 2,207 aa, and a 3’UTR of 71 nt. The whole-genome sequence was compared to the Sabin-2 vaccine reference strain (**Table 1**). Strain SPA866 differed from that of Sabin-2 by 12.7% (846 nt and 66 aa substitutions) in the open reading frame, by 13% (96 nt substitutions) in the 5´UTR and by 4.2% (3 nt substitutions) in the 3´UTR. A key determinant of the Sabin-2 attenuated phenotype at nt 481 of the 5’UTR reverted in strain SPA866 (A-to-G substitution). Nt substitutions were widely distributed throughout the P1 region. A majority (87.5%, 119/136) occurred in the third codon position. Four amino acid (aa) substitutions in the P1 capsid region mapped in virion surface residues form the neutralizing antigenic sites (**Table 1**).

**Table 1:** Nucleotide and amino acid substitutions in strain SPA866 in comparison to Sabin 2.

Genomic region		Nucleotides		Amino acid		Relevant mutations*
		No. substitutions	Divergence (%)	No. substitutions	Divergence (%)	
**5’UTR**		96	13	NA	NA	A481G (Attenuation)
**P1 region**	**VP4**	19	9.2	1	1.4	
	**VP2**	37	4.5	2	0.7	
	**VP3**	34	4.7	4	1.7	Ser73Asn (NAg site 3), Thr75Met (NAg site 3)
	**VP1**	46	5.1	7	2.3	Arg103Lys (NAg site 1), Ile143Thr (Attenuation), Ser222Pro (NAg site 2)
**P2 region**		325	18.8	25	4.3	
**P3 region**		384	17	27	3.6	
**3’UTR**		3	4.2	NA	NA	

*Relevant mutations refer to relevant sites in the Sabin-2 genome described as being involved in Sabin-2 attenuation (Ren et al., 1991) or those predicted neutralizing antigenic (NAg) sites important for immune recognition (Shulman et al., 2000; Adu et al., 2007). NA, not applicable.

### Analysis of recombination events

**Figure 1:**
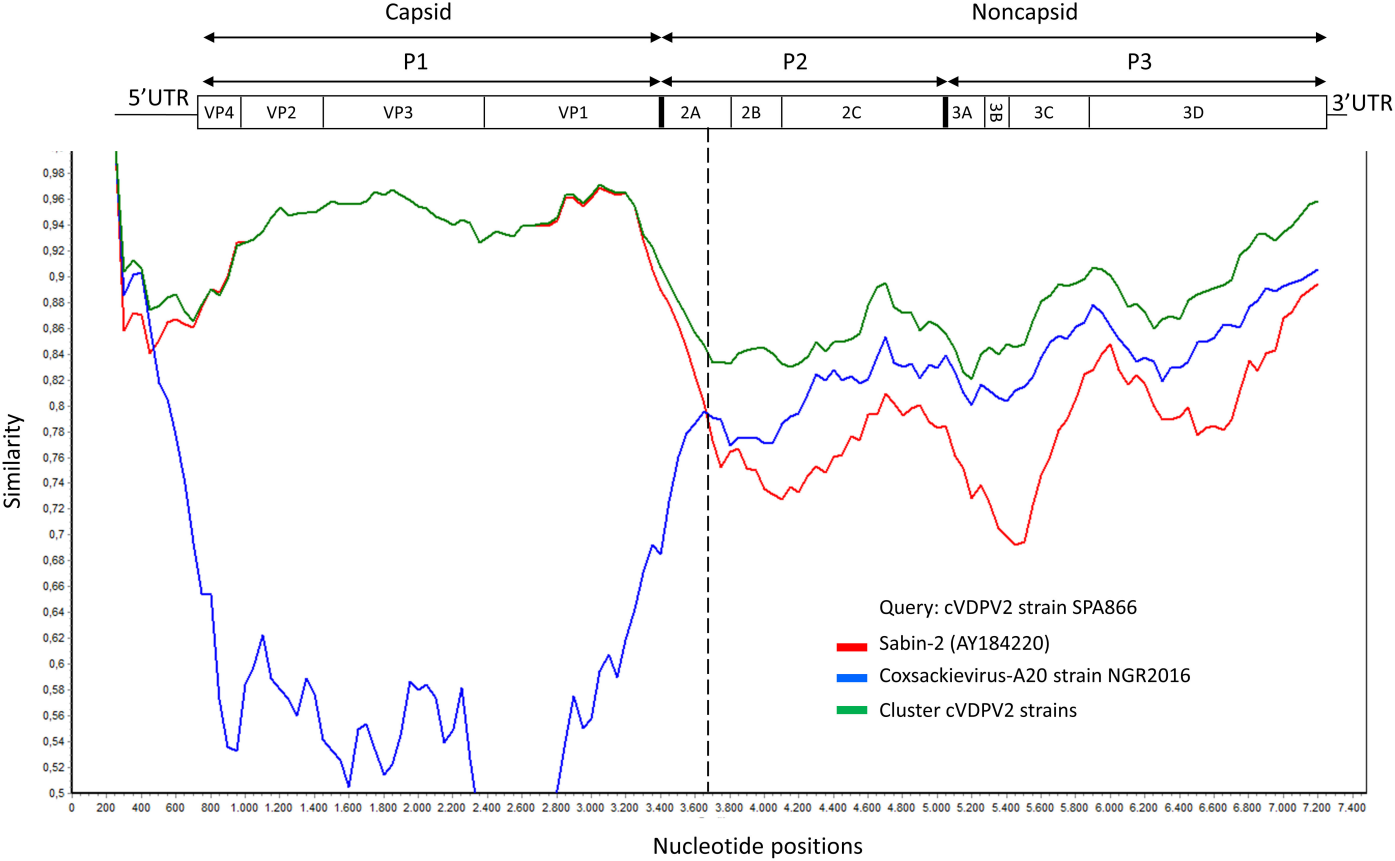
Plot of similarity of whole-genome nucleotide sequences of study strain SPA866 (query), Sabin-2 poliovirus strain, and closely related strains from NCBI. The enterovirus genetic organization is shown in the top panel. The breakpoint position of recombination is marked with a dashed line. cVDPV2 strains: MT432140, MG212462, JX275032, JX275140, JX275015, KJ170572, MG212459, KX162700, KX162714, KX162716, MG212490, MG212480, and MT432143; Coxsackievirus-A20 strain NGR2016: MH785183. cVDPV, type 2 circulating vaccine-derived poliovirus; UTR, untranslated region.

Although the P1 capsid genomic region was similar to that of Sabin-2 (5.1% nt divergence), the nonstructural regions of the genome (P2 and P3) were dissimilar (18.8% and 17% nt divergence, respectively) (**Table 1**). This suggested that the strain SPA866 was a vaccine/non-vaccine recombinant. Similarly, the 5’UTR sequence of SPA866 was apparently derived from non-OPV viruses (13% difference from the Sabin 2 strain). To confirm the existence of recombination events, similarity plot analysis was conducted against complete sequences closely related to strain SPA866 (**Figure 1**). These closely related strains were screened against the NCBI non-redundant nt database using BLASTn (http://www.ncbi.nlm.nih.gov/). The structural P1 coding region and nonstructural P2 and P3 regions of strain SPA866 were used as queries for BLASTn, and sequences with the highest homologies and complete genomes were used in the recombination analysis (**Supplementary Table 1**). A majority of closely related strains found in public databases were type 2 VDPVs from Nigeria, the Central African Republic, Russia, and Ukraine, and only one strain was a non-polio species C enterovirus (coxsackievirus A20 from Nigeria). Similarity plot analysis revealed that the nt similarity in the 3’ half of the genome was <90%. The potential recombination breakpoint was estimated to be in the protease 2A genomic region (position refers to alignment with Sabin-2) (**Figure 1**).

### Phylogenetic analysis

**Figure 2:**
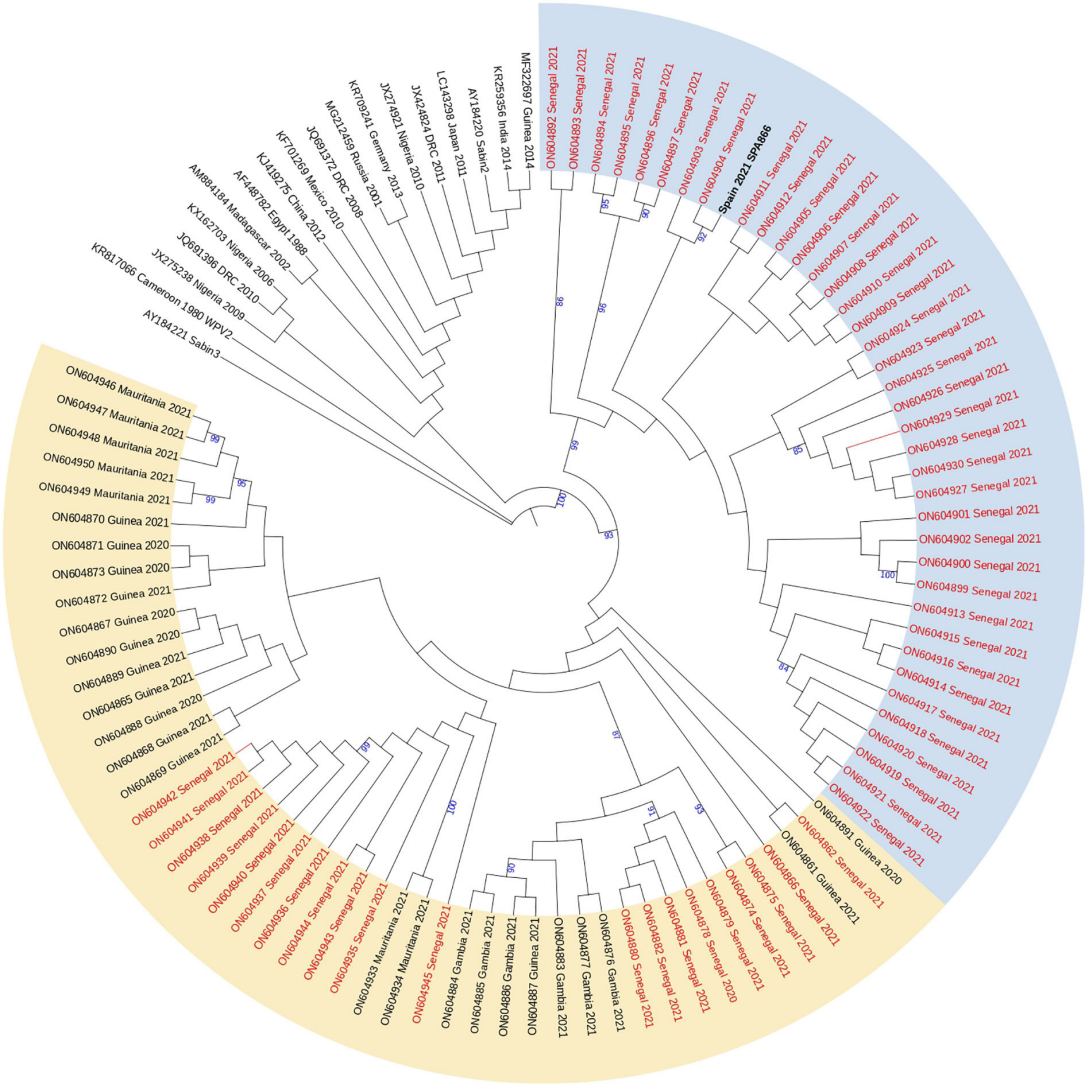
Phylogenetic tree of the complete VP1 coding sequences of VDPV2 study strain SPA866 and 104 sequences from previously described VDPV2 strains extracted from GenBank. The neighbor-joining tree was constructed using MEGA 10.0. Distances were computed using the Kimura 2-parameter model after excluding positions containing gaps and missing data from the alignments. The robustness of the nodes was tested by 1,000 bootstrap replications. Only bootstrap support values above 80% are shown in branch nodes. The scale bar represents nt substitutions per site. The tree is annotated with the classification proposed by Faye et al. for cVDPVs circulating in Senegal since their first introduction in 2020 (clade 1 in yellow and clade 2 in blue). Strains from Senegal are marked in red for clarity. The study strain is marked in bold black. The Sabin-3 poliovirus sequence was introduced as an outgroup.

Analysis of complete VP1 coding sequences showed that strain SPA866 branched closely (bootstrap value of 98%) with cVDPV2 strains obtained in Senegal in 2021, more precisely with those strains previously defined in clade 2 by Faye et al (Faye. et al., 2022). that comprised sequences from Dakar, Diourbel, Thiès, and Louga. Clade 2 strains from Senegal and strain SPA866 were highly similar (nt identity ≥97.2%; aa identity ≥96.7%) supporting that all are genetically related and have a common precursor (**Figure 2**). Strain SPA866 showed the closest relationship to strain ON604904 (bootstrap value of 92%, nt identity = 99.3%, aa identity = 100%), isolated from an AFP case in 2021 from Bambey, in the Diourbel medical region.

### Estimation of the duration of replication of SPA866

**Figure 3:**
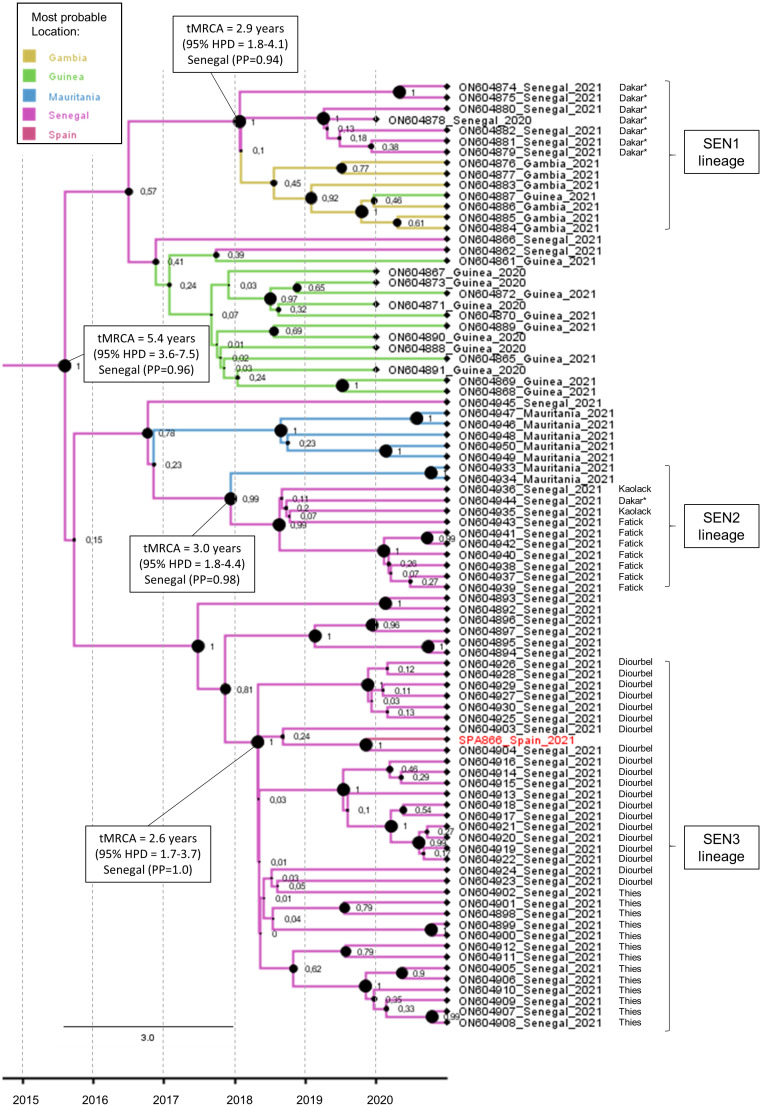
Maximum clade credibility (MCC) subtree of the 86 VP1 coding sequences branching with the SPA866 sequence. Branch colors indicate the most probable location of the MRCA. Node support values are indicated by their values and node size. The scale axis represents the estimated dating of the MRCA for each cluster. The regions within Senegal where samples were taken are indicated. Samples from sewage are indicated by an asterisk.

The maximum clade credibility tree of the complete VP1 coding sequence was estimated for the study strain SPA866 and closely related sequences (clusters 1 and 2) observed in **Figure 2** (**Figure 3**). The sequence SPA866 branched into a lineage (the SEN3 lineage) comprising 34 sequences from samples collected in the Diourbel and Thies regions of Senegal in 2021 (**Figure 3**). The time of the most recent common ancestor (tMRCA) of this lineage is 2.6 years (95% HPD: 1.7–3.7). Two other independent VDPV2 lineage groups were identified with samples from Senegal. The first lineage (SEN1 lineage) comprised sequences from Dakar (Senegal), Guinea, and Gambia, with Senegal being the most likely origin (PP = 0.94) and with a mean tMRCA of 2.9 years (95% HPD: 1.8–4.1). The other lineage (SEN2 lineage) included sequences from southern Senegal (regions of Fatick and Kaolack) and Mauritania, being Senegal the most probable origin (PP = 0.98) and with a mean tMRCA of 3.0 years (95% HPD: 1.8–4.4). These three independent lineages that have arisen approximately at the same time, around early 2018, in different regions of Senegal, share a common ancestor that was circulating in Senegal (PP = 0.96) with a mean tMRCA of 5.4 years (95% HPD: 3.6–7.5). The date of the OPV dose that initiated these three independent lineages observed in Senegal was estimated around 2015, about 5.4 years before 2021 (95% HPD: 3.6–7.5), with robust node support (a posterior probability value of 1).

### Microbiological investigation of case contacts and environmental samples in Spain

All stool samples from 25 close contacts were negative for poliovirus, as were the four concentrated wastewater samples, although rhinoviruses (n = 8) or NPEV of A and B species (n = 6) were detected in some of them (**Supplementary Tables 2, 3**).

### Microbiological investigation of case contacts in Senegal

Field investigations were conducted around the case in the Mbour medical district (Thies medical region) in Senegal. Stool samples collected from 25 contacts tested negative for polioviruses, while 32% of them (n = 8) tested positive for enterovirus B species.

## Discussion

If there are cVDPVs that emerge and circulate due to person-to-person transmission in communities with low polio immunity, there remains the potential for international spread to polio-free countries with high vaccine coverage, such as Spain. Despite the worldwide reported decline in the number of cVDPV2 AFP cases since 2020 (1082 in 2020, 682 in 2021, and 478 in 2022 across 34 countries) (World Health Organization, 2022), the risk of the international spread of cVDPV2 remains quite high. Among these cVDPV2 AFP cases, 59%, 87%, and 72% were reported in Africa in 2020, 2021, and 2022, respectively (World Health Organization, 2022). In 2021, Senegal reported 29 cases of cVDPVs (including 17 paralytic cases), with more than 80% of cVDPV2 being reported in Dakar and Diourbel (UNICEF Senegal, 2022). In this context, in September 2021, a VDPV2 was identified in the stool of a case of AFP admitted to a Spanish hospital from Senegal.

This study describes the isolation and molecular characterization of VDPV2 detected and isolated (SPA866). We demonstrate by sequencing and phylogenetic analysis of the VP1 genomic region that the isolated virus is genetically linked to cVDPVs previously isolated in Diourbel, Senegal. We estimated that the initiating OPV dose for VDPV2s circulating in west Africa in 2020–21 was given around 2015 in Senegal, and it disseminated in early 2018 within the country and lately to nearby countries such as Mauritania, Guinea, and Gambia. Thus, the widespread circulation of cVDPV2s reveals gaps in immunity to PV2 in children across all these countries and their silent circulation until the first detection in wastewater samples in Dakar, Senegal, in December 2020 (Faye. et al., 2022). The Bayesian phylogenetic analysis suggests that the most probable origin of all VDPV2 isolates circulating in Senegal, Guinea, Gambia, and Mauritania was Senegal around 2015. Our results are consistent with previous findings that suggest that VDPV2s in Senegal in 2021 will spread to Gambia (Faye. et al., 2022), given that all VDPV2 strains from the Gambia branch are in the SEN1 lineage, which has Senegal as the most likely origin with a very high probability. Similarly, these results do not contradict the possibility that the origin of some independent VDPV2 introductions observed in Senegal recorded during 2020–21 was Guinea, Mali, and Côte d’Ivoire, as previously suggested (Faye. et al., 2022). It is to be noted that results may be biased given the lack of sequence data from other countries in West Africa.

Enteroviruses are characterized by a great deal of genetic variability, relying on two different evolutionary mechanisms: mutation and recombination (Muslin et al., 2019). In this study, we looked at both mechanisms. Regarding mutations in the complete VP1 coding sequence, we observed that strain SPA866 contained reverse mutations of nt481 (A to G) and nt2909 (Ile to Thr), which are associated with the phenotypic reversion of the attenuated Sabin 2 strain to neurovirulence. Besides mutations, RNA recombination is a molecular process during which genomic fragments from different RNA strands are combined into a single genome (Muslin et al., 2019). RNA recombination with cocirculating NPEV species C (NPEV-C) strains is thought to also contribute to the emergence of cVDPVs with enhanced transmissibility, replication, and/or neurovirulence (Jegouic et al., 2009; Bessaud et al., 2016). Most cVDPVs detected to date have recombinant genomes typically composed of a P1 region (encoding capsid proteins) derived from parental OPV and P2–P3 regions (encoding non-structural proteins) derived from non-vaccine viruses, most likely NPEV-C strains (Kew et al., 2005; Combelas et al., 2011). The NPEV-C sequences found in the P2–P3 regions of previously described pathogenic cVDPVs are CVA17, CVA20, CVA11, and CVA13 (Rakoto-Andrianarivelo et al., 2007; Faleye et al., 2019). In our study, we similarly observed that strain SPA866 may be a vaccine/non-vaccine recombinant given that P2–P3 regions showed high divergences (18.8% and 17%, respectively) with Sabin 2, suggesting that the putative non-OPV sequence of SPA866 was derived from co-circulating NPEV-C. However, the parental NPEV-C strain of the non-capsid sequence could not be identified. This could be due to the scarce number of complete genome sequences for NPEV-C in public databases. For instance, only seven full-genome CVA17 strains were found in GenBank, of which four were from Africa (Madagascar, 2001–2011). Similarly, only one CVA20 from West Africa was found in GenBank (Nigeria, 2016). Another major limitation for recombination analyses was that all NPEV-C sequences retrieved from GenBank were distant both geographically and in time from study strain SPA866 (imported from Senegal, 2021). Future whole-genome sequencing of co-circulating human NPEV-C strains from West African countries, such as Senegal, Guinea, and Mauritania, will provide a clearer picture of the contribution of recombinants to the emergence of West African VDPVs. Vaccine/non-vaccine breakpoints in the genomes of recombinant VDPVs are usually located at the end of the 2A- or 2B-coding sequences, although they may also be found close to the vaccine/non-vaccine junction or in the P3 region (Rakoto-Andrianarivelo et al., 2007; Bessaud et al., 2016; Faleye et al., 2019; Joffret et al., 2021). Here we show that the likely site of recombination of strain SPA866 is in the 2A region, which is in accordance with previous studies describing breakpoints in the genomes of recombinant VDPVs type-2 from Madagascar, Nigeria, and the Central African Republic located at this position (Rakoto-Andrianarivelo et al., 2007; Faleye et al., 2019; Joffret et al., 2021).

A whole-genome sequence might be needed for a higher resolution molecular analysis of poliovirus and is important for the confirmation and classification of a VDPV (Classification and reporting of vaccine-derived polioviruses (VDPV), 2016). For the purposes of poliovirus surveillance, VDPVs are categorized as 1) circulating VDPVs, for which there is evidence of person-to-person transmission in the community, and 2) immunodeficiency-associated VDPVs (iVDPV), which are detected in persons with a primary immunodeficiency (Classification and reporting of vaccine-derived polioviruses (VDPV), 2016). Analysis of whole poliovirus genomes using unbiased metagenomics has important advantages. One is enabling the detection of recombinant genomes. This is useful to facilitate its classification as “circulating” VDPV, given that recombination is favorably favored during person-to-person transmission. Another advantage is the detection of mixtures of poliovirus genomes and genetic variants. This is useful to exclude its classification as iVDPV, given that these are characterized by complex mixtures of poliovirus strains and variants (Classification and reporting of vaccine-derived polioviruses (VDPV), 2016; Montmayeur et al., 2017). In this study, using metagenomics, we observed the recombinant structure of VDPV2 and that no other poliovirus genomes were present in the sample, confirming thus its classification as “circulating” VDPV2.

With our metagenomic protocol, a high percentage of viral reads mapping to the target poliovirus genome per total read (69.5% for stool and 75.8% for isolate) was obtained with a great depth of sequencing coverage (5,931 and 11,581, respectively). This result is in accordance with a previous study that obtained on average 68.5% ± 17.6% of viral reads mapping to reference poliovirus genomes per total read using the same NEB-RNA library kit on poliovirus isolates and a viral RNA kit for nucleic acid extraction (Montmayeur et al., 2017). However, we did not use DNase treatment after nucleic acid extraction to remove host DNA while obtaining highly efficient results and simplifying the steps in the protocol. The use of metagenomic protocols, which are increasingly accessible and affordable, in global poliovirus laboratories during the poliovirus endgame will help not only to expand poliovirus sequencing capacity worldwide but to sequence the whole genome of NPEV-C strains and thus serve in further cVDPV recombination analyses to study their contribution to the emergence of VDPVs.

Polio and NPEVs can remain infectious for as long as 2 months in the sewage. Environmental surveillance by testing wastewater samples is a valuable tool for tracking the distribution of these viruses (Global Polio Eradication Initiative). In Spain, environmental surveillance is not established nationwide but has been conducted in the capital since 1999 to maintain the techniques and protocols to be used when an event of poliovirus importation or an outbreak occurs, such as the one described in this article. Virological results in contacts and wastewater samples contributed to the dismissal poliovirus transmission in the community.

In conclusion, we used a whole-genome sequencing protocol using unbiased metagenomics directly from a clinical sample and from an isolate, obtaining high sequence coverage, efficiency, and throughput. By using this method, we generated the whole genome of an imported VDPV2 in 2021 from Spain. The rates of nt substitutions, the recombinant nature of the genome, and the close genomic linkage with strains from Senegal were consistent with the classification of VDPV2 as a circulating type imported from Senegal. We suggest an ancestral origin in Senegal around 2015 for all VDPV2s circulating in 2020–21 in Senegal, Guinea, Gambia, and Mauritania. Our findings also illustrate the importance of microbiological analysis of environmental samples to rule out further transmission.

## Data availability statement

The datasets presented in this study can be found in online repositories. The names of the repository/repositories and accession number(s) can be found in the article/**Supplementary Material**.

## Ethics statement

Ethical review and approval was not required for the study on human participants in accordance with the local legislation and institutional requirements. Written informed consent to participate in this study was provided by the participants’ legal guardian/next of kin.

## Author contributions

MF-G performed the whole-genome sequencing of the detected VDPV-2 together with phylogenetic and recombination analyses. MC led the National Polio Laboratory and performed the poliovirus virological study of the case and contacts in Spain. FD-F performed the phylogenetic analyses. AM-D led the microbiological laboratory in Murcia. OF and MF performed the microbiological poliovirus study of the case contacts in Senegal. MC-L contributed with the epidemiological data. MG drafted the first version of the manuscript. All authors listed have made a substantial, direct, and intellectual contribution to the work and approved it for publication.
