# Supplementary material for: Metagenomic sequencing, molecular characterization, and Bayesian phylogenetics of imported type 2 vaccine-derived poliovirus, Spain, 2021
Source: Front Cell Infect Microbiol. 2023 May 2;13:1168355. doi: 10.3389/fcimb.2023.1168355 (PMC10185892; doi:10.3389/fcimb.2023.1168355)
Supplement: Supplementary file 1 [file Table_1.docx]

Supplementary Material

Metagenomic sequencing, molecular characterization and Bayesian phylogenetics of imported type 2 Vaccine-Derived Poliovirus, Spain, 2021

Maria Dolores Fernandez-Garcia*, Martin Faye, Francisco Diez-Fuertes, Antonio Moreno-Docón, M. Dolores Chirlaque-López, Ousmane Faye, Maria Cabrerizo

*** Correspondence:** Corresponding Author: mdfernandez@isciii.es

**Supplementary Table 1.** Enterovirus strains with the highest similarity in the nucleotide sequences in genomic regions of the strain SPA866 using BLASTn.

| Genomic region | Type | Strain | % nucleotide identity | Accession number | Genome | Year of sample collection | Country of origin |
| --- | --- | --- | --- | --- | --- | --- | --- |
| P1 | cVDPV2 | IS_001 | 94.92 | MG212490 | complete | - | - |
|  | cVDPV2 | 15763_T2 | 94.92 | MG212459 | complete | 2001 | Russia |
|  | cVDPV2 | NIE1018409 | 94.92 | KJ170572 | complete | 2010 | Nigeria |
|  | cVDPV2 | 18058 | 94.88 | MG212480 | complete | 2002 | Russia |
|  | cVDPV2 | 14732 | 94.88 | MG212462 | complete | 2001 | Ukraine |
| P2 | cVDPV2 | NIE0811456, BAS08-06 | 83.67% | JX275140 | complete | 2008 | Nigeria |
|  | cVDPV2 | CAF-19-RS7-MMP-PGP-005-B6 | 83.56% | MT432143 | complete | 2019 | Central African Republic |
|  | cVDPV2 | CAF-19-RS7-MMP-PGP-005-B3 | 83.56% | MT432140 | complete | 2019 | Central African Republic |
|  | cVDPV2 | NIE0611450, BAS06-01 | 83.45% | JX275015 | complete | 2006 | Nigeria |
|  | CVA20 | NGR_2016 | 83.30% | MH785183 | complete | 2016 | Nigeria |
| P3 | cVDPV2 | NIE1519322 | 87.70% | KX162714 | complete | 2015 | Nigeria |
|  | cVDPV2 | NIE0711199, SOS07-01 | 87.39% | JX275032 | complete | 2007 | Nigeria |
|  | cVDPV2 | NIE0611517 | 87.35% | KX162700 | complete | 2006 | Nigeria |
|  | cVDPV2 | NIE1519325 | 87.19% | KX162716 | complete | 2015 | Nigeria |
|  | CVA20 | NGR_2016 | 86.89% | MH785183 | complete | 2016 | Nigeria |

**Supplementary table 2.** Virological results of stool samples from the 25 studied contacts of the case.

|  | Age (yr) | Stool sample 1 | | | | | | Stool sample 2 | | | | | |
| --- | --- | --- | --- | --- | --- | --- | --- | --- | --- | --- | --- | --- | --- |
|  |  | **ID sample** | **Date** | **RD culture** | **L20B culture** | **EV PCR** | **Result** | **ID sample** | **Date** | **RD culture** | **L20B culture** | **EV PCR** | **Result** |
| 1 | 40 | 42302 | 26-Sep | N | N | N | N | 42303 | 27-Sep | N | N | N | N |
| 2 | 41 | 42309 | 26-Sep | N | N | N | N | 42312 | 27-Sep | N | N | N | N |
| 3 | 8 | 42314 | 26-Sep | N | N | P | RV-A | 42315 | 27-Sep | N | N | P | RV-A |
| 4 | 5 | 42317 | 26-Sep | N | N | N | N | 42319 | 27-Sep | N | N | P | RV-A |
| 5 | 1 | 42324 | 26-Sep | N | N | N | N | 42326 | 27-Sep | N | N | P | RV-A |
| 6 | 32 | 43147 | 28-Sep | N | N | N | N | 44180 | 30-Sep | N | N | P | EV-B |
| 7 | 1 | 43148 | 28-Sep | N | N | N | N | 44173 | 30-Sep | N | N | N | N |
| 8 | 1 | 43149 | 25-Sep | N | N | P | RV-A | 43150 | 27-Sep | N | N | P | RV-A |
| 9 | 3 | 43151 | 29-Sep | N | N | N | N | 43906 | 30-Sep | N | N | P | RV-A |
| 10 | 46 | 43195 | 26-Sep | N | N | P | EV-B | 43196 | 27-Sep | N | N | N | N |
| 11 | 46 | 43198 | 25-Sep | N | N | N | N | 43199 | 26-Sep | N | N | N | N |
| 12 | 40 | 43200 | 26-Sep | N | N | P | EV-B | 43202 | 27-Sep | N | N | N | N |
| 13 | 9 | 43203 | 25-Sep | N | N | N | N | 43204 | 26-Sep | N | N | N | N |
| 14 | 46 | 43205 | 25-Sep | N | N | N | N | 43206 | 26-Sep | N | N | P | EV-B |
| 15 | 64 | 43207 | 26-Sep | N | N | N | N | 43208 | 27-Sep | N | N | N | N |
| 16 | 68 | 43209 | 26-Sep | N | N | N | N | 43210 | 27-Sep | N | N | N | N |
| 17 | 52 | 43211 | 27-Sep | N | N | P | EV-B | 43212 | 28-Sep | N | N | N | N |
| 18 | 65 | 43213 | 26-Sep | N | N | N | N | 43214 | 27-Sep | N | N | N | N |
| 19 | 7 | 43215 | 25-Sep | N | N | N | N | 43216 | 27-Sep | N | N | N | N |
| 20 | 5 | 43217 | 25-Sep | N | N | N | N | 43218 | 27-Sep | N | N | P | RV-A |
| 21 | 56 | 43219 | 25-Sep | N | N | N | N | 43220 | 26-Sep | N | N | N | N |
| 22 | 49 | 43221 | 26-Sep | N | N | N | N | 43222 | 27-Sep | N | N | P | EV-A |
| 23 | 5 | 43223 | 25-Sep | N | N | N | N | 43224 | 26-Sep | N | N | N | N |
| 24 | 23 | 43395 | 30-Sep | N | N | N | N | 43396 | 3-Oct | N | N | N | N |
| 25 | 31 | 44184 | 29-Sep | N | N | N | N | 44185 | 30-Sep | N | N | N | N |

N, negative; P, positive; EV-B, enterovirus especie B; EV-A, enterovirus especie A; RV-A, rhinovirus especie A.

**Supplementary Table 3.** Virological results of wastewater samples analyzed.

| ID sample | Collection date | RD cell culture | LB20 cell culture | EV PCR detection | Result |
| --- | --- | --- | --- | --- | --- |
| A1.15 | 15-Sep | N | N | P | E-3 |
| A2.15 | 15-Sep | N | N | P | EV-B |
| A1.20 | 20-Sep | N | N | P | EV-A |
| A2.20 | 20-Sep | N | N | P | EV-A |

N, negative; P, positive; E-3, echovirus 3; EV-B, enterovirus especie B; EV-A, enterovirus especie A.
